# Semi-Preparative Separation, Absolute Configuration, Stereochemical Stability and Effects on Human Neuronal Cells of MDPV Enantiomers

**DOI:** 10.3390/molecules28052121

**Published:** 2023-02-24

**Authors:** Ana Sofia Almeida, Bárbara Silva, João Pedro Silva, José Augusto Pereira, Fernando Remião, Carla Fernandes

**Affiliations:** 1 Laboratório de Química Orgânica e Farmacêutica, Departamento de Ciências Químicas, Faculdade de Farmácia, Universidade do Porto, Rua Jorge Viterbo Ferreira n° 228, 4050-313 Porto, Portugal; 2 Centro Interdisciplinar de Investigação Marinha e Ambiental (CIIMAR), Universidade do Porto, Terminal de Cruzeiros do Porto de Leixões, Avenida General Norton de Matos, s/n, 4450-208 Matosinhos, Portugal; 3 UCIBIO—Applied Molecular Biosciences Unit, REQUIMTE, Laboratory of Toxicology, Department of Biological Sciences, Faculty of Pharmacy, University of Porto, Rua de Jorge Viterbo Ferreira n° 228, 4050-313 Porto, Portugal; 4 Associate Laboratory i4HB—Institute for Health and Bioeconomy, Faculty of Pharmacy, University of Porto, Rua Jorge Viterbo Ferreira n° 228, 4050-313 Porto, Portugal; 5 ICBAS, Instituto de Ciências Biomédicas de Abel Salazar, Universidade do Porto, Rua Jorge Viterbo Ferreira n° 228, 4050-313 Porto, Portugal

**Keywords:** absolute configuration, electronic circular dichroism, enantioresolution, enantioselectivity, liquid chromatography, MDPV, racemization, SH-SY5Y cells, synthetic cathinones

## Abstract

Synthetic cathinones, such as 3,4-methylenedioxypyrovalerone (MDPV), are widely abused due to their psychostimulant effects. As they are chiral molecules, studies of their stereochemical stability (racemization can occur in certain temperatures and acidic/basic environments) and of their biological and/or toxicity effects (enantiomers might display different properties) are of great relevance. In this study, the liquid chromatography (LC) semi-preparative enantioresolution of MDPV was optimized to collect both enantiomers with high recovery rates and enantiomeric ratio (e.r.) values. The absolute configuration of the MDPV enantiomers was determined by electronic circular dichroism (ECD) with the aid of theoretical calculations. The first eluted enantiomer was identified as *S*-(-)-MDPV and the second eluted enantiomer was identified as *R*-(+)-MDPV. A racemization study was performed by LC-UV, showing enantiomers’ stability up to 48 h at room temperature and 24 h at 37 °C. Racemization was only affected by higher temperatures. The potential enantioselectivity of MDPV in cytotoxicity and in the expression of neuroplasticity-involved proteins—brain-derived neurotrophic factor (BDNF) and cyclin-dependent kinase 5 (Cdk5)—was also evaluated using SH-SY5Y neuroblastoma cells. No enantioselectivity was observed.

## 1. Introduction

The use of new psychoactive substances (NPSs), which are being sold as legal alternatives to illicit drugs [1,2], has been increasing since the 2000s. These compounds quickly became a public health threat, due to their easy accessibility online and in smartshops, with limited information available about their biological and toxicological properties. Moreover, the actual composition of many NPSs is uncertain, which means that consumers might purchase and use them without knowing what they are taking or in what quantities [2,3].

Synthetic cathinones are one of the most reported groups of NPSs worldwide [4,5]. They are β-keto phenethylamine derivatives of cathinone, which is an alkaloid found in khat (*Catha edulis*) leaves. Cathinone is structurally identical and similar in action to amphetamine [6,7]. Synthetic cathinones are widely abused, due to their psychostimulant effects, replacing other highly consumed drugs such as 3,4-methylenedioxymethamphetamine (MDMA) and cocaine [8]. They became available via the Internet and in smartshops and other drug paraphernalia stores, where they were commonly found as “bath salts” under names such as Bloom, Ivory Wave, Vanilla Sky, Blue Silk, and Purple Wave [6,9]. In addition, synthetic cathinones were also known as “legal highs”, “food plants”, “research chemicals”, and other words of description [10].

The cathinone scaffold is easily modified by the addition of several substituents at different positions to obtain new derivatives that can circumvent the law. Thus, new derivatives are continuously emerging on the drug market with unknown properties [11,12].

Furthermore, synthetic cathinones are chiral molecules, meaning that they can exist in two enantiomeric forms, which can present different biological and toxicological properties [13]. Although synthetic cathinones have been widely investigated, studies reporting the influence of stereochemistry on their properties are still limited. Nevertheless, investigation in this area has been growing and enantioselectivity was found in some cases [14,15].

**Figure 1:**
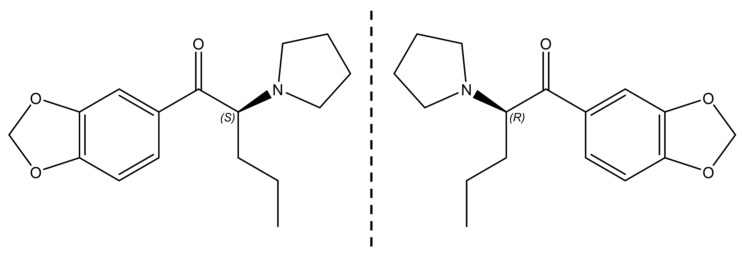
Structures of MDPV enantiomers.

To perform enantioselectivity studies, both forms of enantiomers, of high enantiomeric purity, are needed [16,17]. High purity can be achieved through the resolution of a racemate into the individual enantiomers [18]. Several methods have been described over the years for the analytical enantiomeric resolution of synthetic cathinones [14,15], including capillary electrophoresis [19,20], capillary electrochromatography [21,22], and gas chromatography [23,24]. However, the most-reported technique has undoubtedly been liquid chromatography (LC) using chiral stationary phases (CSPs) [14]. In addition to other advantages, the great number of different types of CSPs is one of the reasons that justify this trend [25,26]. Our group previously reported the analytical enantioresolution of nine synthetic cathinones by LC using different types of CSPs [27]. Moreover, we also performed the semi-preparative separation of the enantiomers of 3,4-methylenedioxypyrovalerone (MDPV) [27] (Figure 1), pentedrone, and methylone by LC using amylose-based CSPs [28].

As an example of a study performed with the individual enantiomers, Kolanos et al. [29] described the *S*-enantiomer of MDPV as displaying greater potency as a reuptake inhibitor of dopamine (DAT) and norepinephrine transporters (NET) and as a facilitator of intracranial self-stimulation (ICSS). Moreover, Gannon et al. [30] reported that *S*-MDPV is predominantly responsible for the effects of the racemate on locomotor activity and core temperature. Our group reported enantioselectivity between the enantiomers of MDPV in permeability studies across the gastrointestinal tract [31]. Additionally, we performed several in vitro studies with the enantiomers of pentedrone and methylone. *R*-(-)-pentedrone and *S*-(-)-methylone were found to be the most permeable compounds across the gastrointestinal tract [32]. Regarding the metabolic profile, *R*-(+)-methylone and *R*-(-)-pentedrone were found to be the most metabolized enantiomers [33]. Enantioselectivity was also observed for these two cathinones on SH-SY5Y neuroblastoma cells, the *S*-(+)-pentedrone and the *R*-(+)-methylone, which were the most oxidative and cytotoxic enantiomers [16].

Neuroplasticity is the ability of the nervous system to modify its neuronal structure or function in response to external and internal changes that emerge from, for instance, development or learning. Since drugs of abuse produce long-lasting effects, neuronal plasticity has been involved in their formation and maintenance [34,35].

Brain-derived neurotrophic factor (BDNF) is part of the neurotrophin family of growth factors and displays multiple roles in the central nervous system (CNS), including the role as a key regulator of synaptic plasticity [36,37]. Due to this role, BDNF was also found to be involved in the actions of drugs of abuse, primarily psychostimulants. Several studies reported a variation (generally an increase) in BDNF expression in different brain regions, mostly in reward-related areas; for example, an increase in BDNF expression was found in rats after acute or repeated cocaine exposure [38,39,40,41].

Synthetic cathinones present psychostimulant properties, and MDPV has been reported to display cocaine-like effects by blocking DAT and NET; however, unlike cocaine, MDPV has no effect on serotonin transporters (SERT) [42]. Thus, investigating the potential role of BDNF in the effects of synthetic cathinones could provide some information about their mechanism of action.

Previous studies reported the effects of MDPV in the expression of BDNF in vivo. Caffino et al. [42] studied the effects of a single exposure to MDPV and α-pyrrolidinovalerophenone (α-PVP) in the expression of BDNF in the brain of the adult mouse. In the frontal lobe, MDPV, but not α-PVP, increased total BDNF mRNA levels at early time points, while after 24 h, both drugs upregulated BDNF mRNA levels. On the other hand, in the hippocampus, both cathinones increased BDNF mRNA levels 30 min and 2 h after exposure, but this effect vanished after 24 h. Moreover, Duart-Castells et al. [43] found that an acute or repeated exposure to MDPV increased cortical BDNF mRNA in mice, while mBDNF protein levels were decreased in the nucleus accumbens 2 h after repeated exposure.

More recently, Nadal-Gratacós et al. [44] showed that after an acute administration of several synthetic cathinones in mice, a tendency to increase BDNF expression was displayed, but only *N*-ethyl-pentylone (NEP) promoted a significant increase of BDNF mRNA levels [44].

Cyclin-dependent kinase 5 (Cdk5) is a serine threonine kinase protein reported to be involved in several aspects of structural and functional neuroplasticity [45]. Cdk5 has been linked to cellular and physiological effects of drug addiction, playing a potential key role in the action of drugs of abuse [46]. Many studies reported changes in the expression of Cdk5 after cocaine exposure in different brain areas [47,48,49]. However, up to now, only two studies related to the effects of MDPV in Cdk5 expression have been reported [50,51]. Duart-Castells et al. [51] reported a significant increase in Cdk5 expression in MDPV-treated mice, with an overexpression still apparent after 3 weeks of withdrawal. However, more recently, another study showed that, although an increase in the expression of ΔFosB (a transcription factor for which Cdk5 has been identified as a target) was observed in MDPV-treated mice, the expression of Cdk5 was not altered 24 h after a conditioned place-preference experiment with MDPV [50]. There is thus a need for a more comprehensive investigation of the effects of MDPV in BDNF and Cdk5 expression.

Herein, we describe the potential enantioselectivity effects of MDPV (Figure 1), one of the most abused synthetic cathinones worldwide [10], on SY-SY5Y human neuroblastoma cells. Specifically, in cytotoxicity and in the expression of BDNF and Cdk5, by a Western-blot analysis. We also optimized the chromatographic conditions for semi-preparative enantioresolution of MDPV by LC to obtain both single enantiomers with high enantiomeric purity and recovery rates. Moreover, the absolute configuration of each enantiomer was determined by electronic circular dichroism (ECD) with the aid of theoretical calculations. Furthermore, to evaluate the stability of the enantiomers in different temperatures and basic conditions, a racemization study was performed by LC with the enantiomers over 48 h.

These studies aim to increase the knowledge about the stereochemical characterization and stability of the MDPV enantiomers and the role of stereochemistry in their biological and/or toxicity effects.

## 2. Results

### 2.1. Semi-Preparative Enantioresolution of MDPV and Evaluation of the Enantiomeric Purity

The MDPV enantiomers were separated in a semi-preparative scale on a homemade column of tris-3,5-dimethylphenylcarbamate amylose coated onto aminopropylsilyl Nucleosyl (500 Å, 7 µm, 20%, *w*/*w*) [52], based on a previous work [27]. After optimization of the chromatographic conditions, a mixture of hexane:ethanol:diethylamine (Hex:EtOH:DEA, 97:3:0.1 *v*/*v*/*v*) as the mobile phase and a flow rate of 1.5 mL/min were used.

**Figure 2:**
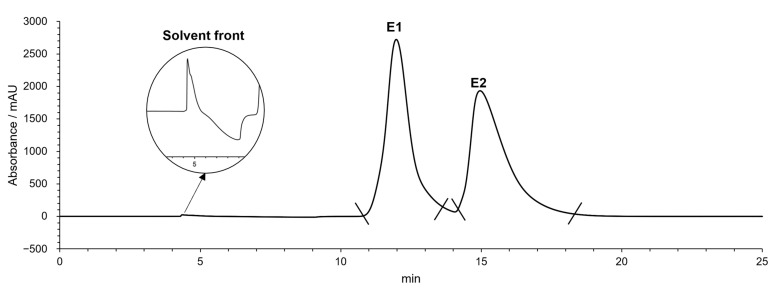
Chromatogram of semi-preparative separation of the enantiomers of MDPV. Chromatographic conditions: tris-3,5-dimethylphenylcarbamate amylose homemade column; mobile phase: Hex:EtOH:DEA (97:3:0.1 *v*/*v*/*v*); flow rate: 1.5 mL/min; UV detection: 254 nm.

The enantiomers of MDPV were successfully separated by this method with a retention time (*t_R_*) of 12.0 min for the first enantiomer (E1) and 15.0 min for the second enantiomer (E2) (Figure 2). An enantioselectivity factor (α) of 1.4 and resolution factor (R_s_) of 1.7 were obtained.

In this work, solutions of 10 mg/mL of racemic MDPV were prepared in EtOH and injections of 100 µL were performed to obtain the isolated enantiomers. The resulting enantiomeric fractions of several injections were combined, and the solvent was evaporated. A total of 110 mg of racemic MDPV were injected in this process after several cycles of injections. Hydrochloride formation was performed using 2 M HCl on diethyl ether. In the end, 50.6 mg of E1 and 51.1 mg of E2 were obtained in hydrochloride form. Recovery rates were calculated using the mass of racemic MDPV injected and the mass obtained for each enantiomer after hydrochloride formation by considering an initial 50:50 proportion in the injected racemate, leading to recovery rates of 92% for E1 and 93% for E2.

**Figure 3:**
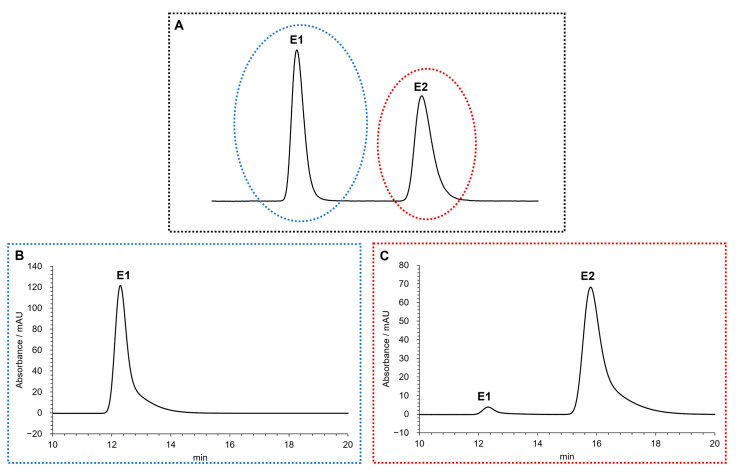
Chromatographs of MDPV racemate (**A**), E1 (**B**) and E2 (**C**). Chromatographic conditions: Lux Amylose-I^®^ column; mobile phase: Hex:EtOH:DEA (97:3:0.1 *v*/*v*/*v*); flow rate: 1 mL/min; UV detection: 254 nm.

For the evaluation of the enantiomeric purity, the same mobile phase was used in an analytical version of the column, specifically the commercial Lux Amylose-I^®^ column. The flow rate was decreased to 1 mL/min and only 10 µL from the solutions in a concentration of 50 µg/mL were used in each injection. First, to determinate the t_R_ of each enantiomer in analytical conditions, a racemic solution of MDPV was injected (Figure 3A), then each enantiomeric fraction. For the first enantiomeric fraction (Figure 3B), an enantiomeric ratio (e.r.) of >99.9% was considered for E1. For the second enantiomeric fraction (Figure 3C), a small peak around 12 min with an area of 3.2 was found corresponding to E1 and a higher peak around 16 min with an area of 61.8 was found corresponding to E2. An e.r. value of 95% was achieved for this enantiomer.

### 2.2. Determination of the Absolute Configuration of the Enantiomers of MDPV

**Figure 4:**
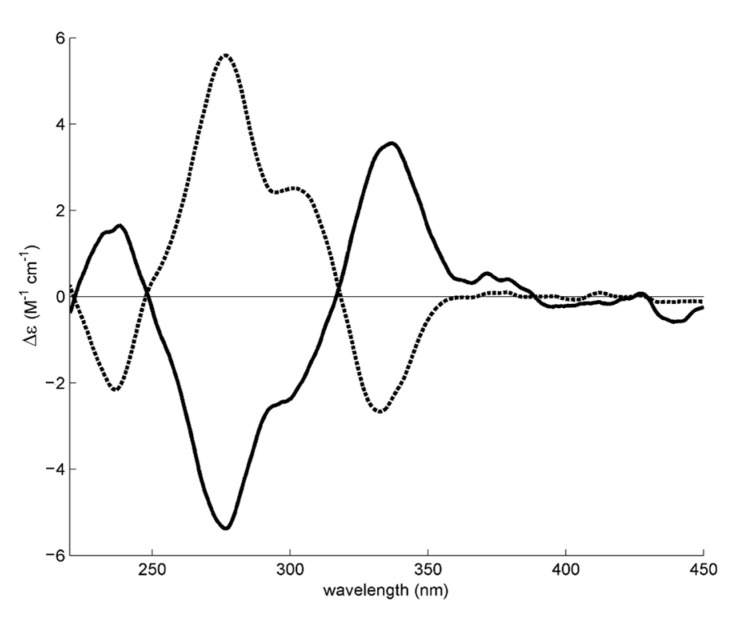
Experimental methanol ECD spectra of MDPV fraction E1 (solid line) and fraction E2 (dashed line). As there was a difference in concentration between the ECD samples, the intensity of the E2 fraction spectrum was amplified 2.5 times for better comparison.

The absolute configuration of MDPV in fraction E1 and E2 was determined by comparing experimental ECD spectra with quantum-mechanical simulations derived from the most significant conformations of the computational models of *S*-MDPV and *R*-MDPV. Figure 4 compares the experimental spectra of the two fractions and suggests that the two MDPV enantiomers were completely separated, as the two spectra are symmetric.

**Figure 5:**
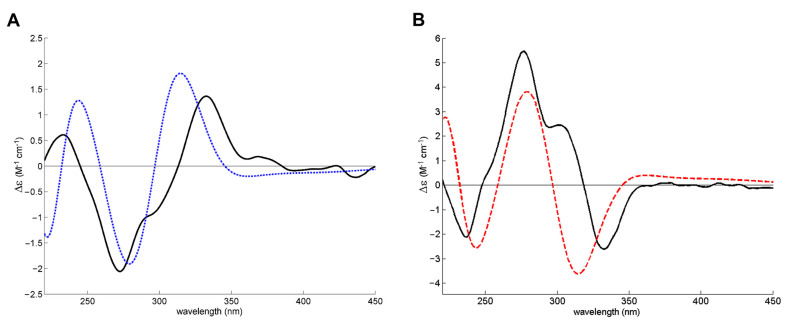
Experimental methanol ECD spectrum of the enantiomeric fractions (solid black line) of E1 (**A**) and E2 (**B**) superimposed with the theoretical ECD spectrum of *S*-MDPV (dashed blue line) and *R*-MDPV (dashed red line) computational models.

Figure 5 compares the experimental spectrum of each fraction with a calculated spectrum for each enantiomer and shows that E1 contains *S*-MDPV and E2 contains *R*-MDPV.

Furthermore, based on our previous work describing the determination of the specific rotation of each enantiomeric fraction [27], the correspondence of E1 to *S*-(-)-MDPV and E2 to *R*-(+)-MDPV was confirmed.

### 2.3. Racemization Study

In this work, the effects of temperature and basic conditions on the racemization of the enantiomers of MDPV were evaluated and analyzed through LC. The analytical chromatographic conditions were identical to those described previously for the evaluation of the enantiomeric purity (Figure 3).

In order to evaluate the effects of temperature in the racemization of the enantiomers of MDPV, the individual enantiomers were exposed to different temperatures: room temperature (RT), 37 °C, and 70 °C. Injections were first performed every 30 min for the first 3 h, then after 24 h and 48 h.

**Figure 6:**
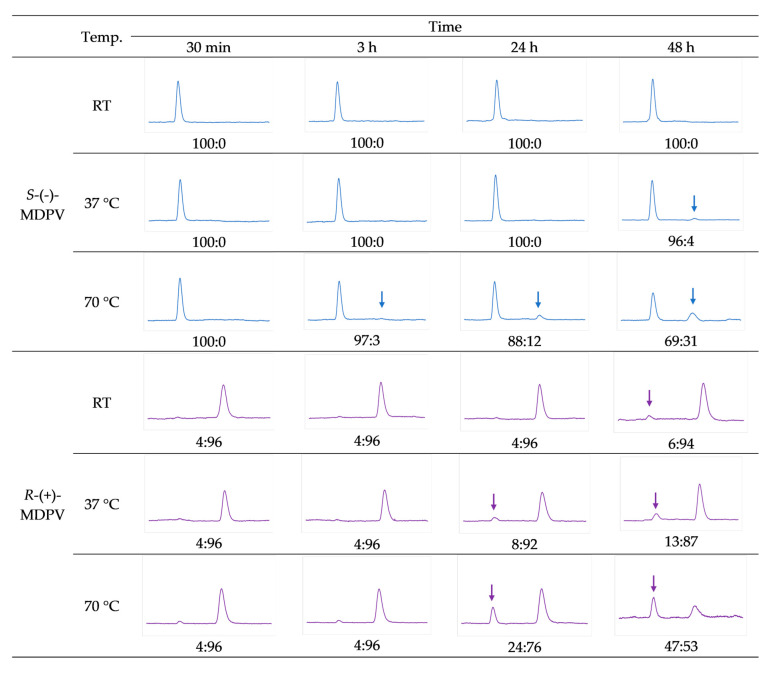
Chromatograms from the racemization study after exposing the enantiomers to different temperatures (RT, 37 °C, and 70 °C), in the absence of DIPEA for 48 h, and respective e.r. values.

The results are presented in Figure 6. These results are expressed as the proportion of *S*-(-)-MDPV:*R*-(+)-MDPV for each chromatogram to better display the changes in the e.r. values. As no significant changes in the e.r. values were observed during the 3 h, only chromatograms from the first time point (30 min) and the last time point (3 h) are shown.

After analyzing the chromatograms corresponding to *S*-(-)-MDPV, no signs of racemization were observed for 48 h at RT and 24 h at 37 °C. After exposing *S*-(-)-MDPV for 48 h to 37 °C, a small peak showed up in the chromatogram with the same *t_R_* as *R*-(+)-MDPV, suggesting that racemization was starting to occur. A change in the e.r. from 100:0 to 96:4 was observed. After 3 h at 70 °C, a change in the e.r. from 100:0 to 97:3 was also observed. The area of *S*-(-)-MDPV increased after 24 h at 70 °C (e.r. of 88:12) and even more after 48 h (69:31).

Regarding the results of *R*-(+)-MDPV, it is important to mention that a small peak corresponding to *S*-(-)-MDPV was observed in every chromatogram, as *R*-(+)-MDPV presented a minor contamination from *S*-(-)-MDPV after separation (e.r. value of 95%) (Figure 3C). Thus, the e.r. of 4:96 was not considered as a sign of racemization for *R*-(+)-MDPV. For *R*-(+)-MDPV, after 48 h at RT and 24 h at 37 °C, there were already changes in the e.r., from 4:96 to 6:94 and 8:92, respectively, which were not observed for *S*-(-)-MDPV. Although no significant change was observed after 3 h at 70 °C, after 24 h the e.r. changed to 24:76 and after 48 h almost full racemization had occurred (47:53).

**Figure 7:**
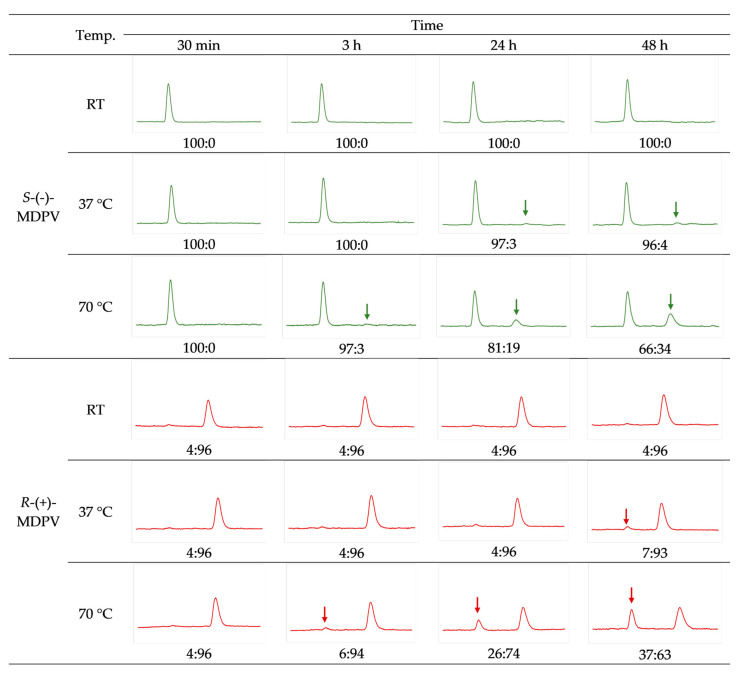
Chromatograms from the racemization study after exposing the enantiomers to different temperatures (RT, 37 °C, and 70 °C), in the presence of DIPEA for 48 h, and respective e.r. values.

To evaluate the effect of basic conditions in the racemization of the enantiomers of MDPV, 0.1% of diisopropylethylamine (DIPEA) was added to the samples and the studies were repeated in the same conditions of temperature as previously. The results are presented in Figure 7.

For *S*-(-)-MDPV, generally, the results were similar both in the presence and absence of DIPEA, with only a few exceptions. For instance, after 24 h at 37 °C, changes in the e.r. were already observed (from 100:0 to 97:3). However, after 48 h, the ratio had no significant change (from 97:3 to 96:4) and was identical to the ratio observed in the absence of DIPEA (96:4). Moreover, after 24 h at 70 °C, in the presence of DIPEA, the decrease in the area of *S*-(-)-MDPV was higher (81:19) than it was in the absence of DIPEA (88:12). Nonetheless, after 48 h, the difference between the ratio in the presence (66:34) and absence (69:31) of DIPEA was smaller.

While for *R*-(+)-MDPV in the absence of DIPEA, small changes in the e.r. were observed after 48 h at RT and 24 h at 37 °C, in the presence of 0.1% DIPEA, no signs of racemization were observed. On the other hand, signs of racemization were observed in this case after 3 h at 70 °C, with a change in the e.r. from 4:96 to 6:94. Moreover, after 48 h at 70 °C in the presence of DIPEA, the decrease in the area of *R*-(+)-MDPV was lower (37:63) than it was in the absence of DIPEA (47:53). The remaining results were similar to the previous results in the absence of DIPEA.

### 2.4. Biological/Toxicological Activity of MDPV Enantiomers in SH-SY5Y Cells

#### 2.4.1. Effects in Cytotoxicity

To evaluate the potential enantioselectivity of the enantiomers of MDPV in cytotoxicity in the SH-SY5Y human neuroblastoma cell line, the 3-(4,5-dimethylthiazol-2-yl)-2,5-diphenyl tetrazolium bromide (MTT) [53] and 3-amino-7-dimethylamino-2-methylphenazine hydrochloride (Neutral Red or NR) [54] assays were performed.

In this work, three concentrations of MDPV were selected, based on Valente et al.’s study [55]: 0.773, 1.165, and 1.506 mM (estimated EC_10_, EC_30_, and EC_50_ in differentiated SH-SY5Y cells). Undifferentiated and dopaminergic SH-SY5Y cells were exposed to the individual enantiomers of MDPV in these concentrations for 24 h.

**Figure 8:**
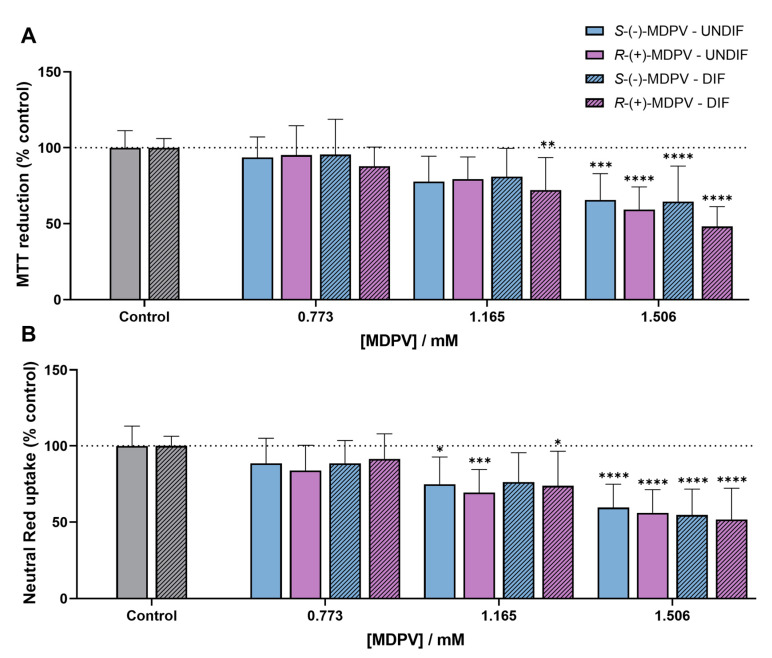
Reduction of MTT (**A**) and NR uptake (**B**) in undifferentiated (UNDIF) and differentiated (DIF) SH-SY5Y cells exposed to the individual enantiomers of MDPV in three concentrations (0.773; 1.165, and 1.506 mM) for 24 h. Results are expressed as mean ± standard deviation (SD) obtained from four independent experiments, run in triplicate. * *p* < 0.05, ** *p* < 0.01, *** *p* < 0.001, **** *p* < 0.0001 vs. respective control samples (undifferentiated or differentiated cells without treatment).

The results (Figure 8) showed that both enantiomers caused a decrease in metabolic activity (Figure 8A) and lysosome integrity (Figure 8B) in a concentration-dependent manner. Significant cytotoxicity (*p* < 0.001) with decreases in cell viability of approximately 50% were observed for the highest concentration (1.506 mM) when compared with control samples (cells without treatment), these results being consistent with that expected for the EC_50_. Significant decreases were also observed for 1.165 mM in some cases (*p* < 0.05). No statistically significant differences were found between the enantiomers in both assays for both cell types (no enantioselectivity). Additionally, no statistically significant difference was found between undifferentiated and differentiated cells when exposed to the enantiomers of MDPV.

#### 2.4.2. Effects in the Expression of Proteins Involved in Neuroplasticity

**Figure 9:**
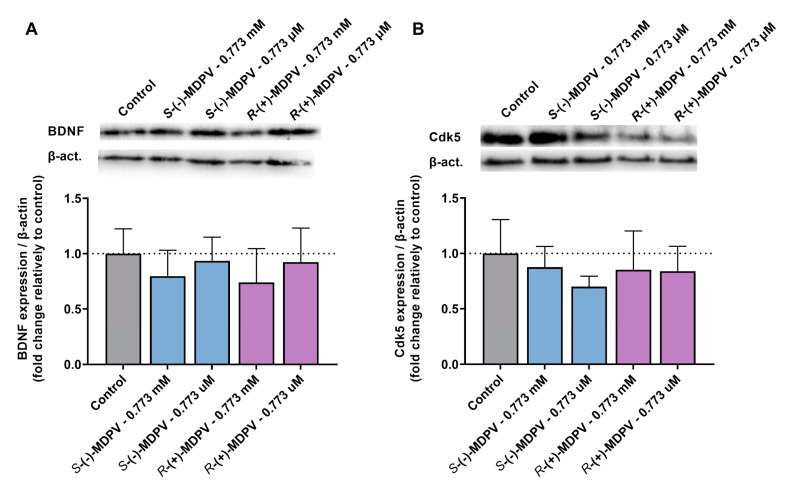
Effects of the enantiomers of MDPV in BDNF (**A**) and Cdk5 (**B**) expression in undifferentiated SH-SY5Y cells. The expression was normalized by β-actin. Results are expressed as mean ± SD for the fold change relative to control samples for at least four independent experiments (*n* = 4) performed in duplicate.

To investigate the effects of the enantiomers of MDPV in the expression of BDNF and Cdk5, proteins involved in neuroplasticity, undifferentiated SH-SY5Y cells were exposed to 0.773 µM and 0.773 mM of each enantiomer and a Western blot analysis was performed. The results are shown in Figure 9.

For BDNF (Figure 9A), although a decrease was observed in the expression of BDNF after exposing SH-SY5Y cells to 0.773 mM of the enantiomers of MDPV, no statistically significant difference was found between MDPV and control samples (cells with no treatment). Additionally, there was no statistically significant difference between the enantiomers in the expression of BDNF for both concentrations (no enantioselectivity). For Cdk5, the results (Figure 9B) were similar to those reported for BDNF.

## 3. Discussion

LC methods have been highly reported to separate the enantiomers of synthetic cathinones [14]. However, most studies only describe their analytical resolution and, since individual enantiomers are needed to perform enantioselectivity studies, scaling up these methods is of great importance.

In a previous work, our group performed, for the first time, the semi-preparative enantioresolution of the enantiomers of MDPV using an amylose-based CSP and Hex:EtOH:triethylamine (TEA) (97:3:0.1 *v*/*v*/*v*) as the mobile phase [27]. A further procedure of extraction was required for TEA removal, which, consequently, decreased the recovery rates of the enantiomers. To avoid this, the chromatographic conditions were optimized. First, a mixture of Hex:EtOH (97:3 *v*/*v*) was selected as the mobile phase and TEA (0.2%) was added to the sample solution of racemic MDPV. Unfortunately, after a few injections, it was found that the presence of TEA caused the degradation of MDPV. Then, DEA was selected as the basic additive and added to the mobile phase. No additive was used in the sample’ solutions. This strategy proved to be effective, as DEA, having a lower boiling point than TEA, was easily eliminated by evaporation under reduced pressure, along with the rest of the mobile phase, and no extra steps were necessary for its removal from the collected samples.

The optimized conditions allowed a successful enantioseparation of MDPV (Figure 2) with good resolution (R_s_ of 1.7) and enantioselectivity (α of 1.4). As the enantiomers were collected in their free form, which is unstable, hydrochloride formation was performed to return the enantiomers to their stable form. High recovery rates (92% and 93%) were obtained.

After this, the enantiomeric purity was determined under analytical chromatographic conditions, reaching e.r. values of >99.9% for E1 and 95% for E2. The lower e.r. value of E2, compared to E1, can be justified by the tailing peak of the first enantiomer.

The absolute configurations of both enantiomers of MDPV were determined by ECD with the aid of theoretical calculations (Figure 4 and Figure 5). Although their absolute configurations have been determined by single-crystal X-ray diffraction [56], the literature shows some inconsistency in the correspondence between the dextrorotatory (+) and levorotatory (-) nomination and the *R* and *S* designation. For example, while Kolanos et al. [29], Schindler et al. [57], Gannon et al. [30], and Aldubayyan et al. [58] used *S*-(+)-MDPV and *R*-(-)-MDPV, Suzuki et al. [56] and Silva et al. [27] considered *S*-(-)-MDPV and *R*-(+)-MDPV. Nonetheless, it is important to mention that the (+)/(-) designation of enantiomers can change if the compound is in a free-base form or a salt, but the *R* or *S* designation will be constant [30]. This could explain the disagreement found in these studies. In a previous work, we determined the (+)/(-) designation of MDPV enantiomers in their hydrochloride form by specific rotation, which resulted in identifying the first collected enantiomer as (-)-MDPV and the second as (+)-MDPV [27]. Herein, the results obtained, for the determination of the absolute configuration of the enantiomers of MDPV in their hydrochloride form, by ECD with the aid of theoretical calculations confirmed the following correspondence: *S*-(-)-MDPV for E1 and *R*-(+)-MDPV for E2.

Considering that enantiomers can racemize in certain conditions of temperature and acidic/basic environments [59], studying their stereochemical stability in various conditions is of great importance. In fact, some drugs, such as ibuprofen and thalidomide, undergo in vivo chiral inversion or racemization, meaning that one or both of the enantiomers are converted into the other in the body [60,61]. Thus, even if the enantiomers are successfully separated, studying their individual properties might be difficult if they undergo racemization. Many studies have reported the application of LC methods to study the racemization of a variety of chiral compounds [59,62,63]. Recently, Aldubayyan et al. [58] studied the potential chiral inversion of the enantiomers of MDPV. The results showed that the *R*-enantiomer undergoes inversion to *S*-MDPV after 24 h at 37 °C and RT in whole blood and methanolic solution to a high degree, while very slight change was observed for *S*-MDPV [58].

Herein, a racemization study was performed for the enantiomers of MDPV in different temperatures and basic conditions by LC using an amylose-based CSP. The individual enantiomers were exposed to different temperatures: RT, which was the work temperature for all the separation process; 37 °C, which was the physiological temperature (also used for biological assays); and 70 °C, which was a more extreme temperature. Due to the short half-life of MDPV (77.8–97.9 min in rats) [64], analyses were first performed closer in time to each other (every 30 min) for the first 3 h to evaluate the stereochemical stability of the enantiomers during that time period. Moreover, injections were performed after 24 and 48 h, which are common time points used for biological assays.

After analyzing the chromatograms (Figure 6 and Figure 7), the enantiomers were considered to be stable for 48 h at RT and 24 h at 37 °C, as minor/no changes were observed in the e.r. values. However, the racemization of the enantiomers of MDPV was affected by the increase in temperature and the time of analysis, as after 48 h at 37 °C and 24 h at 70 °C, a higher degree of racemization was observed. Furthermore, *R*-(+)-MDPV seemed to undergo racemization to a higher degree and faster than *S*-(-)-MDPV. For instance, while for *S*-(-)-MDPV no changes in the e.r. values were observed after 48 h at RT and 24 h at 37 °C, for *R*-(+)-MDPV small changes were observed at those time points (from 4:96 to 6:94 and 8:92, respectively). Moreover, at 70 °C, a higher degree of racemization was also observed for *R*-(+)-MDPV after 24 and 48 h. As previously mentioned, the samples of *R*-(+)-MDPV had in their composition a small amount of *S*-(-)-MDPV (Figure 3C), which could facilitate racemization and explain the differences between enantiomers. Nonetheless, Aldubayyan et al. [58] suggested that the process of inversion from *S* to *R*-MDPV occurs more slowly than from *R* to *S*-MDPV, which could also explain our results. Furthermore, it is important to mention that, in comparison with the previous work, where a high degree of racemization was observed for *R*-MDPV for 24 h at RT and 37 °C, in our study, this enantiomer remained stable. This could be explained by the use of different solvents (whole blood and methanol in the previous study vs. EtOH in our study). In fact, Aldubayyan et al. [58] reported no chiral inversion when using acetonitrile as the solvent.

The racemization study was repeated in basic conditions by the addition of DIPEA to the samples. DIPEA was selected, based on a previous racemization study [62]. Synthetic cathinones are less stable in their base forms [65], so increasing the pH could also lead to a higher rate of racemization for the enantiomers. The results showed no difference or minor differences when comparing the e.r. obtained in the presence of DIPEA with the e.r. obtained in the absence of DIPEA, suggesting that DIPEA has no considerable effect on the racemization of the enantiomers of MDPV. The main differences were found for *S*-(-)-MDPV after 24 h at 70 °C (81:19 in the presence of DIPEA vs. 88:12 in the absence of DIPEA) and *R*-(+)-MDPV after 48 h at 70 °C (47:53 in the presence of DIPEA vs. 37:63 in the absence of DIPEA).

To evaluate the potential enantioselectivity of the enantiomers of MDPV in cytotoxicity, the MTT [53] and NR [54] assays were performed in the SH-SY5Y human neuroblastoma cell line. This cell line was chosen because it is a widely used in vitro model system for a variety of neurotoxicity assays, including the assessment of synthetic cathinones’ neurotoxicity [55,66,67], and presents many advantages compared to other models, such as being rapidly cultured in large quantities with inexpensive maintenance. Additionally, its human origin helps to avoid inter-species differences [68]. A major advantage of SH-SY5Y cells for studies with synthetic cathinones and, specifically MDPV, is their catecholaminergic characteristics expressing DAT and NET, the main targets of MDPV [8,69]. The cytotoxic effects of racemic MDPV in the SH-SY5Y cell line were previously reported in both undifferentiated and dopaminergic SH-SY5Y cells [55].

Valente et al. [55] reported that, when SH-SY5Y cells were differentiated into a dopaminergic phenotype, they display more susceptibility to the cytotoxicity effects of β-keto amphetamines, including racemic MDPV, when compared to undifferentiated SH-SY5Y cells [55]. Moreover, in another study, enantioselective differences were found in cytotoxicity for the enantiomers of two synthetic cathinones, pentedrone and methylone, in dopaminergic SH-SY5Y cells [70]. Thus, these assays were used not only to determine if the enantiomers displayed different effects (enantioselectivity) in the same cell type, but also to compare the effects of the same enantiomer between undifferentiated and differentiated cells, which were previously reported to be different for racemic MDPV [55].

The results (Figure 8) showed that exposing the cells to 1.165 and 1.506 mM of the enantiomers of MDPV caused significant cytotoxic effects, with a higher decrease in cell viability for the highest concentration. Moreover, the percentages of cell viability obtained for each concentration were consistent with percentages expected for EC_10_, EC_30_, and EC_50_ (similar to 90%, 70%, and 50%, respectively). No enantioselectivity was observed for MDPV in the assays, as there were no significant differences between enantiomers. Additionally, no statistically significant difference was found between undifferentiated and differentiated cells when exposed to the enantiomers of MDPV.

Furthermore, the same cell line was used to evaluate the effects of MDPV and potential enantioselectivity in the expression of proteins involved in neuroplasticity, BDNF and Cdk5. Literature on the effects of MDPV in the expression of BDNF and Cdk5 is still scarce. Nonetheless, based on those studies, an alteration in the expression of BDNF after MDPV exposure could be expected [42,43]. For Cdk5, the two studies reported to date showed distinct results [50,51]. Thus, the potential effect of MDPV in Cdk5 expression is still unclear. Undifferentiated SH-SY5Y cells were selected for this assay, as there is no need for extra differentiation steps; therefore, the cells could be exposed to the enantiomers in the following day to seeding, allowing a quicker data acquisition. Moreover, 0.773 mM was selected as one of the concentrations, as low cytotoxicity was observed for this concentration in SH-SY5Y cells (Figure 8). A concentration much lower than 0.773 µM was also selected. The results showed that the enantiomers of MDPV, in the experimental conditions, have no effect in the expression of BDNF or Cdk5 in undifferentiated SH-SY5Y cells (Figure 9), suggesting that MDPV has no interference in the biological processes regulated by these proteins. Additionally, no enantioselectivity was observed.

## 4. Materials and Methods

### 4.1. Reagents and Samples

Racemic MDPV was purchased from the Sensearomatics website (www.sensearomatics.eu, accessed on 19 July 2019. Hex and EtOH for HPLC, DEA, TEA, HCl solution (2 M in diethyl ether), Dulbecco’s modified Eagle’s medium (DMEM) with 4.5 g/L glucose, Triton X-100, MTT, and NR solutions were obtained from the Sigma–Aldrich Co (St. Louis, MO, USA). Hank’s balanced salt solution with or without calcium and magnesium [HBSS (+/+) or HBSS (-/-), respectively], fetal bovine serum (FBS), penicillin (10,000 U/mL)/streptomycin (10,000 μg/mL) solution, and 0.25% trypsin/1 mM EDTA solution were purchased from Gibco Laboratories (Lenexa, KS, USA). Unless stated otherwise, other chemicals of analytical grade were obtained from Merck (Darmstadt, Germany).

### 4.2. Sample Preparation

For the semi-preparative enantioresolution, racemic MDPV was dissolved in EtOH to afford a concentration of 10 mg/mL.

For the racemization study, fractions of each enantiomer were dissolved in EtOH to afford a concentration of 0.5 mg/mL. Twelve vials, six from each enantiomer, were prepared by diluting the previous solutions to afford a concentration of 50 µg/mL and, to three vials from each enantiomer, DIPEA (0.1%) was added. A set of vials from each enantiomer, one with amine and one without amine, were exposed to different temperatures: RT, 37 °C, and 70 °C.

### 4.3. Instrumentation and Chromatographic Conditions

#### 4.3.1. Semi-Preparative Enantioseparation

A Thermo^®^ Scientific HPLC (Thermo Fisher Scientific Inc., Waltham, MA, USA) composed by a Thermo^®^ Scientific Spectra System UV8000 model DAD, P4000 pump and AS3000 automatic injector was selected. A homemade CSP of tris-3,5-dimethylphenylcarbamate amylose coated onto aminopropylsilyl Nucleosyl (500 Å, 7 µm, 20%, *w*/*w*) and packed into a stainless steel column (20 cm × 7.0 mm ID) was used to perform the semi-preparative enantioseparation [52]. The analyses were carried out in normal phase elution mode with Hex:EtOH:DEA (97:3:0.1 *v*/*v*/*v*) as the mobile phase. The mobile phases were degassed in an ultrasonic bath (Sonorex Digitec, Bandelin) for at least 15 min before use. The flow rate used was 1.5 mL/min. Cycles of 2 injections of 100 µL of racemic MDPV were intercalated with one injection of 50 µL of EtOH being the duration of each run 25 min. Chromatographic analyses were performed in isocratic mode at 25 ± 2 °C, with UV detection at 254 nm. ChromeleonTM 7.0 software (Thermo Fisher Scientific Inc., Waltham, MA, USA) was used to process the chromatographic data.

After collecting, the enantiomeric fractions were placed in a round bottom flask and the solvent was evaporated under reduced pressure. Then, hydrochloride formation was performed by precipitation on ice with 2 M HCl on diethyl ether.

#### 4.3.2. Enantiomeric Purity Evaluation and Racemization Study

A JASCO model 880-PU pump HPLC apparatus with a Rheodyne model 7125 injector equipped with a 20 μL sample loop, a JASCO model 880-30 solvent mixer and a JASCO model 875-UV detector (Tokyo, Japan) was used. Data were obtained using ChromNAV.

Chromatographic analyses were performed on a Lux Amylose-I^®^ column (tris-3,5-dimethylphenylcarbamate amylose bonded to 5 µm particle size; 25 cm, 4.6 mm I.D.) from Phenomenex, Inc., Torrance, CA, USA. Injections (10 µL) were performed every 30 min for 3 h and after 24 h and 48 h for each condition, with a flow rate of 1 mL/min. The analyses were performed in normal phase elution mode with a mixture of Hex:EtOH:DEA (97:3:0.1 *v*/*v*/*v*) as the mobile phase. Chromatographic analyses were performed in isocratic mode at 25 ± 2 °C with UV detection at 254 nm. Each run (20 min) was monitored, and e.r. values were calculated for each chromatogram (Equation (4)).

### 4.4. Chromatographic Parameters Determination

The retention factor (*k*) was obtained using Equation (1):

(1) $$k=\frac{t_{R}-t_{0}}{t_{0}}\;$$

The dead time (*t*_0_) was considered to be the peak of the solvent front. α was calculated using Equation (2):

(2) $$\alpha =\frac{k_{2}}{k_{1}}\;$$

An α equal to or above 1.1 indicates selectivity. Furthermore, the following equation was used to calculate the R_s_:

(3) $$R_{s}=1.18\frac{t_{R2}-t_{R1}}{W_{1^{0.5}}+W_{2^{0.5}}}$$

where *t*_*R*1_ and *t*_*R*2_ are the *t_R_* of the first and second enantiomers, respectively, and *W*_1 0.5_ and *W*_2 0.5_ are the corresponding peak widths measured on half height. R_s_ equal to or above 1.5 indicates baseline separation.

The e.r. values were determined by the relative percentages of the peak areas [71]:

(4) $$\mathrm{ER}\left(\%\right)=100\;\frac{\left[E1\right]}{\left[E1\right]+\left[E2\right]}\;\mathrm{or}\;100\;\frac{\left[E2\right]}{\left[E1\right]+\left[E2\right]}$$

where [E1] and [E2] are the areas of the peak of each enantiomer.

### 4.5. Determination of the Absolute Configuration of the Enantiomers by ECD

The experimental ECD spectrum of MDPV (2–4 mg/mL in acetonitrile) was obtained in a Jasco J-815 CD spectropolarimeter with a 0.1 mm cuvette and 8 accumulations. The simulated ECD spectra were obtained by first determining all the relevant conformers of the computational model using GaussView’s GMMX module (Gaussian Inc., Wallingford, CT, USA). The resulting 52 molecular mechanics conformers were minimized using the quantum mechanical DFT method B3LYP/6-31G with Gaussian 16W (Gaussian Inc., Wallingford, CT, USA). The lowest energy models, representing 95% of the Boltzmann populations (20 models), were subjected to a final minimization round using the B3LYP/6-311+G(2d,p)/methanol method (Gaussian 16W). The TD B3LYP/6-311+G(2d,p)/methanol method was used to calculate the first 50 transitions of the 7 lowest energy (90% of the population. The line spectrum of each of the 7 models was built by applying a Gaussian line broadening of 0.25 eV to each computed transition. The final ECD spectrum was obtained by the Boltzmann-weighted sum of the 7 line spectra [72]. This whole process was applied to the neutral model, MDPV, and to the protonated model, MDPVH^+^. No significant ECD spectral differences were found between the two models and both final spectra are consistent with the enantiomeric assignment mentioned above.

### 4.6. Cell Culture

The SH-SY5Y human neuroblastoma cell line was acquired from ATCC (American Tissue Culture Collection, Manassas, VA, USA), and routinely maintained in DMEM, supplemented with 10% FBS and 1% penicillin/streptomycin solution, at 37 °C in a humidified atmosphere of 5% CO_2_, and the medium was changed every 2 days. Subcultures were performed by trypsinization with a 0.25% trypsin/EDTA solution once cultures reached about 70–80% confluence.

Cytotoxicity assays were performed in both undifferentiated and dopaminergic-differentiated SH-SY5Y cells. Cells were seeded in 96-well plates at a density of 25,000 cells/cm^2^. SH-SY5Y cell differentiation was induced with 10 μM retinoic acid (RA) for 72 h, followed by 80 nM tetradecanoyl phorbol 13-acetate (TPA) for another 72 h, as previously described, to obtain a dopaminergic phenotype [55]. Undifferentiated cells were allowed to grow during the same time without the addition of differentiation factors. For the preparation of total protein extracts for Western blot analysis, undifferentiated cells were seeded in 6-well plates at a density of 25,000 cells/cm^2^ and left to adhere overnight before exposure. For all assays, cells were used between passages 23 to 31.

### 4.7. Cytotoxicity Assays

#### 4.7.1. MTT Reduction Assay

The cells’ metabolic activity was assessed using the MTT reduction assay, as previously described [73]. This method assesses the reduction of MTT by mitochondrial dehydrogenases and intracellular NADPH-dependent oxidoreductases into formazan crystals. After the differentiation protocol described above, the medium was removed and the cells were exposed to different concentrations of each enantiomer of MDPV (0.773; 1.165 and 1.506 mM [55]), in fresh medium. After 24 h, the medium was removed and an MTT solution (0.5 mg/mL in HBSS(+/+)) was added for 90 min. The MTT solution was replaced with dimethyl sulfoxide (DMSO) to dissolve the formed formazan crystals. Finally, the absorbance was measured at 550 nm in a 96-well plate reader (PowerWaveX; Bio-Tek, Winooski, VT, USA). As the positive control, 1% Triton X-100 was used. Data were expressed as the percentage of metabolic activity relative to negative control samples (untreated cells) from four independent experiments performed in triplicate.

#### 4.7.2. Neutral Red Assay

The NR uptake assay was used, as previously described [73], to evaluate lysosomal integrity. This method assesses the incorporation of NR by the lysosomes from viable cells. After the differentiation protocol, the medium was removed and replaced with different concentrations of each enantiomer of MDPV (0.773; 1.165 and 1.506 mM [56]), in fresh medium. After 24 h, the medium was removed and an NR solution (50 µg/mL in HBSS(+/+)) was added for 90 min. After discarding the NR solution, a lysis solution (EtOH:H_2_O:glacial acetic acid (50:49:1, *v*/*v*/*v*)) was added. Finally, the absorbance was measured at 540 nm in a 96-well plate reader (PowerWaveX; Bio-Tek, Winooski, VT, USA). As the positive control, 1% Triton X-100 was used. Data were expressed as the percentage of NR uptake relative to negative control samples (untreated cells) from four independent experiments performed in triplicate.

### 4.8. Preparation of Total Protein Extracts

After 24 h of exposure to 0.773 µM and 0.773 mM of each enantiomer of MDPV, total protein extracts were prepared. Cells were collected with a Teflon cell scraper and centrifuged at 1000 g for 5 min at 4 °C. Cold HBSS(-/-) was used to wash the pellets before they were centrifuged again for 5 min, at 1000 g and 4 °C. Collecting buffer (1mM EDTA, 2 mM MgCl_2_, 10 mM KCl, 20 mM HEPES, 250 mM Sucrose, pH 7.5) supplemented with a protease inhibitor solution (1 µg/mL of pepstatin A, antipain, leupeptin and chymostatin), 100 µM phenylmethylsulfonyl fluoride (PMSF) and 2 mM dithiothreitol (DTT) was added to resuspend the pellets. Sonication was performed to disrupt the cells, using three 10 s pulses separated by 10 s intervals on ice. Samples were further stored at −80 °C until use. To quantify total protein content, the Bradford method was used.

### 4.9. Western Blot Analysis

Expression of BDNF and Cdk5 was detected through Western blot analysis [73]. A total of 25–30 μg of protein for each sample were denatured in sample loading buffer at 95 °C and loaded onto 10% or 15% acrylamide/bis-acrylamide sodium dodecyl sulphate-polyacrylamide gel electrophoresis (SDS/PAGE). After electrophoresis, the proteins were transferred to nitrocellulose membranes. Then, membranes were blocked in 5% skim milk for 2 h, washed three times in TPBS (phosphate buffered saline, pH 7.4, containing 0.05% Tween 20), and incubated overnight at 4 °C with the following antibodies: anti-Cdk5 antibody (1:200 dilution) (Santa Cruz Biotechnology, CA, USA) and anti-BDNF antibody (1:200) (Santa Cruz Biotechnology, CA, USA). β-actin detection was used as a protein-loading control, by incubation with an anti-β-actin antibody (1:5000) (Sigma–Aldrich, St. Louis, MO, USA). In the following day, the membranes were washed in TPBS and incubated for 1 h with a secondary anti-mouse horseradish peroxidase (HRP) conjugate antibody (1:20,000). Immunoreactive bands were viewed using the chemiluminescent Clarity™ Western ECL substrate and analyzed using the Bio-Rad ChemiDoc™ XRS + System with Image Lab™ Software (Hercules, CA, USA). Band intensities were quantified using ImageLab 6.1 (Bio-Rad, Berkeley, CA, USA) and normalized against the intensities of β-actin. The results were expressed as fold-change relative to the control samples (untreated cells). Full images of the blots (Figures S1 and S2) and densitometry readings/intensity ratios (Table S1) can be found in the Supplementary Materials.

### 4.10. Statistical Analysis

The GraphPad Prism 9.0 software (San Diego, CA, USA) was used to perform all statistical analysis. Normality of data distribution was assessed by the Kolmogorov–Smirnov and Shapiro–Wilk normality tests. To make statistical comparisons, one/two-way analysis of variance (ANOVA) followed by the Tukey’s/Holm–Sidak’s multiple comparisons test were performed. Data were displayed as mean ± SD of the number of independent experiments indicated in each case. Statistically significant differences between values were considered for *p* values lower than 0.05.

## 5. Conclusions

In this work, the semi-preparative separation of the enantiomers of MDPV was successfully performed with good resolution and enantioselectivity, the enantiomers being collected with high e.r. values and recovery rates.

The determination of the absolute configuration of the enantiomers allowed the identification of E1 as *S*-(-)-MDPV and E2 as *R*-(+)-MDPV.

For 48 h at RT and 24 h at 37 °C, the enantiomers were considered stereochemically stable, as no changes or only minor changes were observed in the e.r. values. Nevertheless, higher temperatures can cause a higher degree of racemization. DIPEA had no considerable effect on the racemization of the enantiomers of MDPV.

The enantiomers of MDPV displayed similar concentration-dependent cytotoxicity in the SH-SY5Y cell line (no enantioselectivity was observed). No statistically significant difference was found between undifferentiated and differentiated cells in each condition.

The enantiomers of MDPV had no effect on the expression of the neuroplasticity-involved proteins BDNF and Cdk5 in SH-SY5Y cells and no statistically significant differences were found between the enantiomers.

Although no enantioselectivity was found for MDPV in the performed assays, synthetic cathinones are still highly consumed and new derivatives are continuously emerging with unknown properties. Thus, investigation in this research field is still necessary to better understand the role of stereochemistry in the biological and toxicity properties of this class of compounds.

## Data Availability

Data are contained within the article or supplementary material.

## Supplementary Materials

The following supporting information can be downloaded at: https://www.mdpi.com/article/10.3390/molecules28052121/s1. Figure S1—Whole western blot with molecular weight markers for the results of BDNF (Figure 9A). Figure S2—Whole western blot with molecular weight markers for the results of Cdk5 (Figure 9B). Table S1—Densitometry readings of BDNF, Cdk5 and β-actin and corresponding ratios.

## Sample Availability

Samples of the compounds are available from the authors.

## References

[1] Zanda M.T., Fattore L., Watson R.R., Zibadi S. (2017). Chapter 29—Novel Psychoactive Substances: A New Behavioral and Mental Health Threat. Addictive Substances and Neurological Disease.

[2] Coppola M., Mondola R., Oliva F., Picci R.L., Ascheri D., Trivelli F., Preedy V.R. (2016). Chapter 63—Treating the Phenomenon of New Psychoactive Substances: Synthetic Cannabinoids and Synthetic Cathinones. Neuropathology of Drug Addictions and Substance Misuse.

[3] Zawilska J.B. (2015). Chapter Thirteen—“Legal Highs”—An Emerging Epidemic of Novel Psychoactive Substances. Int. Rev. Neurobiol..

[4] EMCDDA (2020). New Psychoactive Substances: Global Markets, Glocal Threats and the COVID-19 Pandemic—An Update from the EU Early Warning System. https://www.emcdda.europa.eu/publications/rapid-communication/new-psychoactive-substances-global-markets-glocal-threats-and-covid-19-pandemic_en.

[5] Soares J., Costa V.M., Bastos M.d.L., Carvalho F., Capela J.P. (2021). An updated review on synthetic cathinones. Arch. Toxicol..

[6] German C.L., Fleckenstein A.E., Hanson G.R. (2014). Bath salts and synthetic cathinones: An emerging designer drug phenomenon. Life Sci..

[7] Valente M.J., Guedes de Pinho P., de Lourdes Bastos M., Carvalho F., Carvalho M. (2014). Khat and synthetic cathinones: A review. Arch. Toxicol..

[8] Baumann M.H., Partilla J.S., Lehner K.R., Thorndike E.B., Hoffman A.F., Holy M., Rothman R.B., Goldberg S.R., Lupica C.R., Sitte H.H. (2013). Powerful cocaine-like actions of 3,4-methylenedioxypyrovalerone (MDPV), a principal constituent of psychoactive ‘bath salts’ products. Neuropsychopharmacol.

[9] Bretteville-Jensen A.L., Tuv S.S., Bilgrei O.R., Fjeld B., Bachs L. (2013). Synthetic cannabinoids and cathinones: Prevalence and markets. Forensic Sci. Rev..

[10] Protti M., Dasgupta A. (2019). Chapter 21—Review of Bath Salts on Illicit Drug Market. Critical Issues in Alcohol and Drugs of Abuse Testing.

[11] Paillet-Loilier M., Cesbron A., Boisselier R., Bourgine J., Debruyne D. (2014). Emerging drugs of abuse: Current perspectives on substituted cathinones. Subst. Abus. Rehabil..

[12] Kuropka P., Zawadzki M., Szpot P. (2022). A review of synthetic cathinones emerging in recent years (2019–2022). Forensic Toxicol..

[13] Coelho M.M., Fernandes C., Remião F., Tiritan M.E. (2021). Enantioselectivity in Drug Pharmacokinetics and Toxicity: Pharmacological Relevance and Analytical Methods. Molecules.

[14] Almeida A.S., Silva B., Pinho P.G., Remião F., Fernandes C. (2022). Synthetic Cathinones: Recent Developments, Enantioselectivity Studies and Enantioseparation Methods. Molecules.

[15] Silva B., Fernandes C., Guedes de Pinho P., Remião F. (2018). Chiral Resolution and Enantioselectivity of Synthetic Cathinones: A Brief Review. J. Anal. Toxicol..

[16] Francotte E.R. (2001). Enantioselective chromatography as a powerful alternative for the preparation of drug enantiomers. J. Chromatogr. A.

[17] Tiritan M.E., Pinto M., Fernandes C. (2020). Enantioselective Synthesis, Enantiomeric Separations and Chiral Recognition. Molecules.

[18] Pinto M.M.M., Fernandes C., Tiritan M.E. (2020). Chiral Separations in Preparative Scale: A Medicinal Chemistry Point of View. Molecules.

[19] Hägele J.S., Hubner E.M., Schmid M.G. (2019). Chiral separation of cathinone derivatives using β-cyclodextrin-assisted capillary electrophoresis-Comparison of four different β-cyclodextrin derivatives used as chiral selectors. Electrophoresis.

[20] Pérez-Alcaraz A., Borrull F., Aguilar C., Calull M. (2019). Enantioselective determination of cathinones in urine by high pressure in-line SPE-CE. Electrophoresis.

[21] Aturki Z., Schmid M.G., Chankvetadze B., Fanali S. (2014). Enantiomeric separation of new cathinone derivatives designer drugs by capillary electrochromatography using a chiral stationary phase, based on amylose tris(5-chloro-2-methylphenylcarbamate). Electrophoresis.

[22] Albals D., Heyden Y.V., Schmid M.G., Chankvetadze B., Mangelings D. (2016). Chiral separations of cathinone and amphetamine-derivatives: Comparative study between capillary electrochromatography, supercritical fluid chromatography and three liquid chromatographic modes. J. Pharm. Biomed. Anal..

[23] Alremeithi R.H., Meetani M.A., Khalil S.A. (2016). A validated gas chromatography mass spectrometry method for simultaneous determination of cathinone related drug enantiomers in urine and plasma. RSC Adv..

[24] Alremeithi R., Meetani M.A., Alaidaros A.A., Lanjawi A., Alsumaiti K. (2018). Simultaneous Quantitative Determination of Synthetic Cathinone Enantiomers in Urine and Plasma Using GC-NCI-MS. J. Anal. Methods Chem..

[25] Teixeira J., Tiritan M.E., Pinto M.M.M., Fernandes C. (2019). Chiral Stationary Phases for Liquid Chromatography: Recent Developments. Molecules.

[26] Fernandes C., Lima R., Pinto M.M.M., Tiritan M.E. (2022). Chromatographic supports for enantioselective liquid chromatography: Evolution and innovative trends. J. Chromatogr. A.

[27] Silva B., Fernandes C., Tiritan M.E., Pinto M.M., Valente M.J., Carvalho M., de Pinho P.G., Remião F. (2016). Chiral enantioresolution of cathinone derivatives present in “legal highs”, and enantioselectivity evaluation on cytotoxicity of 3,4-methylenedioxypyrovalerone (MDPV). Forensic Toxicol..

[28] Silva B., Pereira J.A., Cravo S., Araújo A.M., Fernandes C., Pinto M.M.M., de Pinho P.G., Remião F. (2018). Multi-milligram resolution and determination of absolute configuration of pentedrone and methylone enantiomers. J. Chromatogr. B.

[29] Kolanos R., Partilla J.S., Baumann M.H., Hutsell B.A., Banks M.L., Negus S.S., Glennon R.A. (2015). Stereoselective Actions of Methylenedioxypyrovalerone (MDPV) To Inhibit Dopamine and Norepinephrine Transporters and Facilitate Intracranial Self-Stimulation in Rats. ACS Chem. Neurosci..

[30] Gannon B.M., Williamson A., Suzuki M., Rice K.C., Fantegrossi W.E. (2016). Stereoselective Effects of Abused “Bath Salt” Constituent 3,4-Methylenedioxypyrovalerone in Mice: Drug Discrimination, Locomotor Activity, and Thermoregulation. J. Pharm. Exp. Ther..

[31] Almeida A.S., Silva B., Remião F., Fernandes C. (2023). Assessment of the Permeability of 3,4-Methylenedioxypyrovalerone (MDPV) across the Caco-2 Monolayer for Estimation of Intestinal Absorption and Enantioselectivity. Int. J. Mol. Sci..

[32] Silva B., Silva R., Fernandes C., Guedes de Pinho P., Remião F. (2020). Enantioselectivity on the absorption of methylone and pentedrone using Caco-2 cell line: Development and validation of an UHPLC method for cathinones quantification. Toxicol. Appl. Pharm..

[33] Silva B., Rodrigues J.S., Almeida A.S., Lima A.R., Fernandes C., Guedes de Pinho P., Miranda J.P., Remião F. (2022). Enantioselectivity of Pentedrone and Methylone on Metabolic Profiling in 2D and 3D Human Hepatocyte-like Cells. Pharmaceuticals.

[34] Castrén E., Antila H. (2017). Neuronal plasticity and neurotrophic factors in drug responses. Mol. Psychiatry.

[35] von Bernhardi R., Bernhardi L.E., Eugenín J. (2017). What Is Neural Plasticity?. Adv. Exp. Med. Biol..

[36] Poo M.M. (2001). Neurotrophins as synaptic modulators. Nat. Rev. Neurosci..

[37] Bramham C.R., Messaoudi E. (2005). BDNF function in adult synaptic plasticity: The synaptic consolidation hypothesis. Prog. Neurobiol..

[38] Filip M., Faron-Górecka A., Kuśmider M., Gołda A., Frankowska M., Dziedzicka-Wasylewska M. (2006). Alterations in BDNF and trkB mRNAs following acute or sensitizing cocaine treatments and withdrawal. Brain Res..

[39] Fumagalli F., Caffino L., Racagni G., Riva M.A. (2009). Repeated stress prevents cocaine-induced activation of BDNF signaling in rat prefrontal cortex. Eur. Neuropsychopharmacol..

[40] Fumagalli F., Moro F., Caffino L., Orrù A., Cassina C., Giannotti G., Di Clemente A., Racagni G., Riva M.A., Cervo L. (2013). Region-specific effects on BDNF expression after contingent or non-contingent cocaine i.v. self-administration in rats. Int. J. Neuropsychopharmacol..

[41] Li X., Wolf M.E. (2015). Multiple faces of BDNF in cocaine addiction. Behav. Brain Res..

[42] Caffino L., Mottarlini F., Bilel S., Targa G., Tirri M., Maggi C., Marti M., Fumagalli F. (2021). Single Exposure to the Cathinones MDPV and α-PVP Alters Molecular Markers of Neuroplasticity in the Adult Mouse Brain. Int. J. Mol. Sci..

[43] Duart-Castells L., López-Arnau R., Vizcaíno S., Camarasa J., Pubill D., Escubedo E. (2019). 7,8-Dihydroxyflavone blocks the development of behavioral sensitization to MDPV, but not to cocaine: Differential role of the BDNF-TrkB pathway. Biochem. Pharm..

[44] Nadal-Gratacós N., Alberto-Silva A.S., Rodríguez-Soler M., Urquizu E., Espinosa-Velasco M., Jäntsch K., Holy M., Batllori X., Berzosa X., Pubill D. (2021). Structure-Activity Relationship of Novel Second-Generation Synthetic Cathinones: Mechanism of Action, Locomotion, Reward, and Immediate-Early Genes. Front. Pharm..

[45] Cortés N., Guzmán-Martínez L., Andrade V., González A., Maccioni R.B. (2019). CDK5: A Unique CDK and Its Multiple Roles in the Nervous System. J. Alzheimers Dis..

[46] Benavides D.R., Bibb J.A. (2004). Role of Cdk5 in drug abuse and plasticity. Ann. N. Y. Acad. Sci..

[47] Bibb J.A., Chen J., Taylor J.R., Svenningsson P., Nishi A., Snyder G.L., Yan Z., Sagawa Z.K., Ouimet C.C., Nairn A.C. (2001). Effects of chronic exposure to cocaine are regulated by the neuronal protein Cdk5. Nature.

[48] Lu L., Grimm J.W., Shaham Y., Hope B.T. (2003). Molecular neuroadaptations in the accumbens and ventral tegmental area during the first 90 days of forced abstinence from cocaine self-administration in rats. J. Neurochem..

[49] Wedzony K., Markowicz-Kula K., Chocyk A., Fijał K., Maćkowiak M. (2005). The effect of ‘binge’ cocaine administration on the expression of cyclin-dependent kinase 5 and its activator p35 in various regions of rat brain. Brain Res..

[50] Duart-Castells L., Blanco-Gandía M.C., Ferrer-Pérez C., Puster B., Pubill D., Miñarro J., Escubedo E., Rodríguez-Arias M. (2020). Cross-reinstatement between 3,4-methylenedioxypyrovalerone (MDPV) and cocaine using conditioned place preference. Prog. Neuropsychopharmacol. Biol. Psychiatry.

[51] Duart-Castells L., López-Arnau R., Buenrostro-Jáuregui M., Muñoz-Villegas P., Valverde O., Camarasa J., Pubill D., Escubedo E. (2019). Neuroadaptive changes and behavioral effects after a sensitization regime of MDPV. Neuropharmacology.

[52] Matlin S.A., Tiritan M.E., Crawford A.J., Cass Q.B., Boyd D.R. (1994). HPLC with carbohydrate carbamate chiral phases: Influence of chiral phase structure on enantioselectivity. Chirality.

[53] Präbst K., Engelhardt H., Ringgeler S., Hübner H. (2017). Basic Colorimetric Proliferation Assays: MTT, WST, and Resazurin. Methods Mol. Biol..

[54] Repetto G., del Peso A., Zurita J.L. (2008). Neutral red uptake assay for the estimation of cell viability/cytotoxicity. Nat. Protoc..

[55] Valente M.J., Bastos M.d.L., Fernandes E., Carvalho F., Guedes de Pinho P., Carvalho M. (2017). Neurotoxicity of β-Keto Amphetamines: Deathly Mechanisms Elicited by Methylone and MDPV in Human Dopaminergic SH-SY5Y Cells. ACS Chem. Neurosci..

[56] Suzuki M., Deschamps J.R., Jacobson A.E., Rice K.C. (2015). Chiral Resolution and Absolute Configuration of the Enantiomers of the Psychoactive “Designer Drug” 3,4-Methylenedioxypyrovalerone. Chirality.

[57] Schindler C.W., Thorndike E.B., Suzuki M., Rice K.C., Baumann M.H. (2016). Pharmacological mechanisms underlying the cardiovascular effects of the “bath salt” constituent 3,4-methylenedioxypyrovalerone (MDPV). Br. J. Pharm..

[58] Aldubayyan A.A., Castrignanò E., Elliott S., Abbate V. (2023). Development and validation of a chiral LC-MS/MS method for the separation and quantification of four synthetic cathinones in human whole blood and its application in stability analysis. Talanta.

[59] Huang Z., Guo D., Fan J., Zhong Y., Zhang M., He L., Zhang W. (2020). HPLC semi-preparative separation of diclazuril enantiomers and racemization in solution. J. Sep. Sci..

[60] Nguyen L.A., He H., Pham-Huy C. (2006). Chiral drugs: An overview. Int. J. Biomed. Sci..

[61] Davies N.M., Teng X.W. (2003). Importance of chirality in drug therapy and pharmacy practice: Implications for psychiatry. Adv. Pharm..

[62] Steber S.E., Pham A.N.D.L., Nelson E., Wolf C. (2021). Enantioseparation and racemization of α-aryl-α-fluoroacetonitriles. Chirality.

[63] Hofmann J., Fayez S., Scheiner M., Hoffmann M., Oerter S., Appelt-Menzel A., Maher P., Maurice T., Bringmann G., Decker M. (2020). Sterubin: Enantioresolution and Configurational Stability, Enantiomeric Purity in Nature, and Neuroprotective Activity In Vitro and In Vivo. Chem. Eur. J..

[64] Anizan S., Concheiro M., Lehner K.R., Bukhari M.O., Suzuki M., Rice K.C., Baumann M.H., Huestis M.A. (2016). Linear pharmacokinetics of 3,4-methylenedioxypyrovalerone (MDPV) and its metabolites in the rat: Relationship to pharmacodynamic effects. Addict. Biol..

[65] Tsujikawa K., Mikuma T., Kuwayama K., Miyaguchi H., Kanamori T., Iwata Y.T., Inoue H. (2012). Degradation pathways of 4-methylmethcathinone in alkaline solution and stability of methcathinone analogs in various pH solutions. Forensic Sci. Int..

[66] Soares J., Costa V.M., Gaspar H., Santos S., de Lourdes Bastos M., Carvalho F., Capela J.P. (2019). Structure-cytotoxicity relationship profile of 13 synthetic cathinones in differentiated human SH-SY5Y neuronal cells. NeuroToxicology.

[67] Valente M.J., Amaral C., Correia-da-Silva G., Duarte J.A., Bastos M.L., Carvalho F., Guedes de Pinho P., Carvalho M. (2017). Methylone and MDPV activate autophagy in human dopaminergic SH-SY5Y cells: A new insight into the context of β-keto amphetamines-related neurotoxicity. Arch. Toxicol..

[68] Strother L., Miles G.B., Holiday A.R., Cheng Y., Doherty G.H. (2021). Long-term culture of SH-SY5Y neuroblastoma cells in the absence of neurotrophins: A novel model of neuronal ageing. J. Neurosci. Methods.

[69] Kovalevich J., Langford D. (2013). Considerations for the use of SH-SY5Y neuroblastoma cells in neurobiology. Methods Mol. Biol..

[70] Silva B., Palmeira A., Silva R., Fernandes C., Guedes de Pinho P., Remião F. (2021). S-(+)-Pentedrone and R-(+)-methylone as the most oxidative and cytotoxic enantiomers to dopaminergic SH-SY5Y cells: Role of MRP1 and P-gp in cathinones enantioselectivity. Toxicol. Appl. Pharm..

[71] Tiritan M.E., Fernandes C., Maia A.S., Pinto M., Cass Q.B. (2018). Enantiomeric ratios: Why so many notations?. J. Chromatogr. A.

[72] Stephens P.J., Harada N. (2010). ECD cotton effect approximated by the Gaussian curve and other methods. Chirality.

[73] Silva J.P., Carmo H., Carvalho F. (2018). The synthetic cannabinoid XLR-11 induces in vitro nephrotoxicity by impairment of endocannabinoid-mediated regulation of mitochondrial function homeostasis and triggering of apoptosis. Toxicol. Lett..

