# Supplementary material for: Semi-Preparative Separation, Absolute Configuration, Stereochemical Stability and Effects on Human Neuronal Cells of MDPV Enantiomers
Source: Molecules. 2023 Feb 24;28(5):2121. doi: 10.3390/molecules28052121 (PMC10003790; doi:10.3390/molecules28052121)
Supplement: Supplementary file 1 [file molecules-28-02121-s001.zip › molecules-2234875-supplementary.pdf]

# Supplementary Material

*Article*

# Semi-Preparative Separation, Absolute Configuration, Stereochemical Stability and Effects on Human Neuronal Cells of MDPV Enantiomers

**Ana Sofia Almeida [1,2,3,4], Bárbara Silva [3,4], João Pedro Silva [3,4], José Augusto Pereira [2,5], Fernando Remião [3,4,*] and Carla Fernandes [1,2,*]**

[1] Laboratório de Química Orgânica e Farmacêutica, Departamento de Ciências Químicas, Faculdade de Farmácia, Universidade do Porto, Rua Jorge Viterbo Ferreira n° 228, 4050-313 Porto, Portugal
[2] Centro Interdisciplinar de Investigação Marinha e Ambiental (CIIMAR), Universidade do Porto, Terminal de Cruzeiros do Porto de Leixões, Avenida General Norton de Matos, s/n, 4450-208 Matosinhos, Portugal
[3] UCIBIO—Applied Molecular Biosciences Unit, REQUIMTE, Laboratory of Toxicology, Department of Biological Sciences, Faculty of Pharmacy, University of Porto, Rua de Jorge Viterbo Ferreira n° 228, 4050-313 Porto, Portugal
[4] Associate Laboratory i4HB—Institute for Health and Bioeconomy, Faculty of Pharmacy, University of Porto, Rua Jorge Viterbo Ferreira n° 228, 4050-313 Porto, Portugal
[5] ICBAS, Instituto de Ciências Biomédicas de Abel Salazar, Universidade do Porto, Rua Jorge Viterbo Ferreira n° 228, 4050-313 Porto, Portugal
* Correspondence: remiao@ff.up.pt (F.R.); cfernandes@ff.up.pt (C.F.)

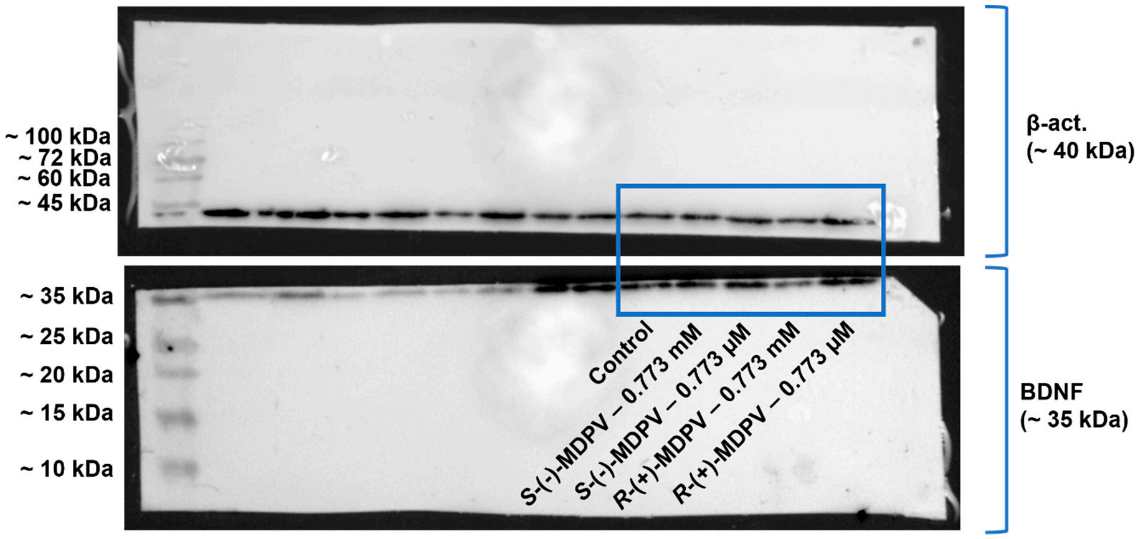


**Figure S1**. Whole western blot with molecular weight markers for the results of BDNF (Figure 9A).

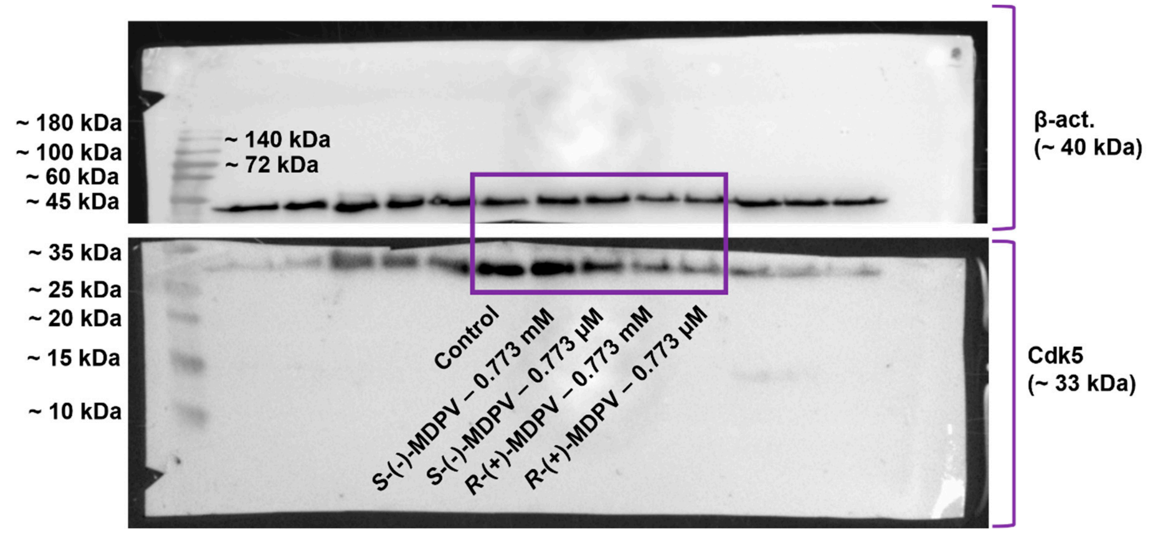


**Figure S2.** Whole western blot with molecular weight markers for the results of Cdk5 (Figure 9B).

**Table S1.** Densitometry readings of BDNF, Cdk5 and β-actin and corresponding ratios.

| | **BDNF** | **β-act.** | **BDNF/ β-act.** | **Cdk5** | **β-act.** | **Cdk5/ β-act.** |
|---|---|---|---|---|---|---|
| **Control** | 7114721 | 10102257 | 0.754 | 6665473 | 10697999 | 0.732 |
| ***S*-(-)-MDPV – 0.773 mM** | 3812395 | 7248132 | 0.600 | 5794052 | 8519039 | 0.640 |
| ***S*-(-)-MDPV – 0.773 μM** | 7222052 | 10555884 | 0.704 | 6455587 | 13709127 | 0.512 |
| ***R*-(+)-MDPV – 0.773 mM** | 3815989 | 7868410 | 0.558 | 3666241 | 8042054 | 0.624 |
| ***R*-(+)-MDPV – 0.773 μM** | 6552368 | 9517177 | 0.696 | 5232219 | 10429950 | 0.615 |
